# Pre-operative Status of Gut Microbiota Predicts Post-operative Delirium in Patients With Gastric Cancer

**DOI:** 10.3389/fpsyt.2022.944236

**Published:** 2022-07-07

**Authors:** Meiru Jiang, Wenfei Tan

**Affiliations:** Department of Anesthesiology, The First Hospital of China Medical University, Shenyang, China

**Keywords:** gut microbiota (GM), post-operative delirium (POD), 16S rRNA, principle coordinate analysis (PCoA), PCA, gastric cancer

## Introduction

We read with great interest the recent article by Liu et al. (1) and congratulate the authors for their innovative analysis of the pre-operative gut microbiota between patients with and without post-operative delirium (POD). This article included very important clinical data for early diagnosis and determination of the treatment for POD. However, there are some important points of concern.

First, we wonder about the sample size. Research on the correlation between gut microbiota and perioperative cognitive dysfunction, especially for humans, is still rare. Hence, the method used to determine the minimum sample size is yet to be ascertained. Therefore, using the distribution of that metric in published but related studies is the first step to estimating the sample size (2). Since we found that related studies included over 80 sample sizes (3) to increase the reliability of the results in most cases, we are concerned that only 20 fecal samples in each cohort may be unable to completely show the differences in gut microbiota between the two cohorts.

Moreover, all the patients in this trial underwent general anesthesia, but Hu Liu et al. limited the drugs in anesthesia induction and post-operation without during the operation-which could influence the morbidity of post-operative delirium significantly (4). Thus, we suggest that increasing the sample size or limiting the anesthetics during operation might higher the qualification of the results if the author chose not to determine the use of anesthetics during the operation.

Second, we are also curious about the incomplete β-diversity analysis results in Figure 2. We noticed that the author used Principal Component Analysis (PCA) in Figure 2A and Principal Co-ordinates Analysis (PCoA) in Figure 2B but missed the percent of PCA 1 and PCoA 1 and their quotiety of explanation on the X-axis equally and marked the PCA 2 and PCoA 2 on the Y-axis only, which should not be omitted (5). We expect the author to add these analyses in Figure 2 for the readers.

Furthermore, we searched the registration number (ChiCTR200030131) on the Chinese Clinical Trial Registry given by the author but failed to find any registered trail. We hope that the author could proofread this to confirm.

Our research team has been studying the “brain-gut” axis and submitted one similar study on clinicaltrails.gov in 2020 (NCT04316910), but it was unfortunate that the plan has been delayed because of funding issues. However, Hu Liu et al. derived a more original research study compared to ours. Therefore, we are very excited to read this article and sincerely hope that Hu Liu et al. can achieve greater academic success in this field in the future.

## Author Contributions

WT contributed to the conception and the review of the manuscript. MJ wrote the first draft and contributed to the editing of the manuscript. Both authors contributed to manuscript revision, read, and approved the submitted version.

## Conflict of Interest

The authors declare that the research was conducted in the absence of any commercial or financial relationships that could be construed as a potential conflict of interest.

## Publisher's Note

All claims expressed in this article are solely those of the authors and do not necessarily represent those of their affiliated organizations, or those of the publisher, the editors and the reviewers. Any product that may be evaluated in this article, or claim that may be made by its manufacturer, is not guaranteed or endorsed by the publisher.
